# Chronic Pain Management in Primary Care: A Review of Practical, Individualized Approaches for Clinical Practice

**DOI:** 10.3390/jpm16100507

**Published:** 2026-09-29

**Authors:** Ahmad Mirza, Imran Gani, Shameem Beigh

**Affiliations:** Department of Medicine, Wellstar MCG Health, Augusta University, Augusta, GA 30912, USA

**Keywords:** chronic pain, personalized medicine, pain phenotyping, primary care, biopsychosocial model, multimodal treatment, patient-centered care, primary care workflow

## Abstract

**Background:** Chronic pain is a complex condition affecting approximately 20–30% of adults worldwide. Increasing evidence demonstrates that chronic pain is a heterogeneous condition influenced by biological mechanisms, psychological factors, social circumstances and individual patient characteristics. These differences contribute to variable treatment responses and highlight the need for individualized clinical care. **Objective:** This narrative review provides a practical, clinical framework for individualized chronic pain management in primary care, emphasizing mechanism-informed clinical phenotyping, non-pharmacological and pharmacological interventions, shared decision-making, continuous reassessment and practical implementation within time-constrained practice settings. **Methods:** A non-systematic literature search of MEDLINE/PubMed, the Cochrane Database of Systematic Reviews and current international clinical guidelines (e.g., IASP, ICD-11, NICE, and CDC) was conducted for contemporary literature published from 2015 to 2026. Evidence was qualitatively selected and synthesized regarding pain mechanisms, primary care assessment, non-pharmacological therapies, modern pharmacotherapy, and health implementation frameworks. **Results:** Individualized chronic pain care requires the identification of contributing clinical pain mechanisms—nociceptive, neuropathic, nociplastic or mixed—while recognizing that these exist on a continuum rather than as mutually exclusive entities. Effective primary care management prioritizes active non-pharmacological interventions (exercise, pain neuroscience education as part of multidisciplinary rehabilitation, psychological therapies and sleep optimization) followed by selective, mechanism-informed pharmacotherapy. Regular reassessment using multidimensional functional criteria is essential for the optimization of benefit while minimizing treatment-related harms. **Conclusion:** Primary care clinicians are well positioned to deliver individualized chronic pain management through longitudinal relationships, clinical phenotyping, physical examination and coordinated multimodal care. An individualized approach focused on patient-defined functional goals provides a practical model for improving outcomes in primary care.

## 1. Introduction

Chronic pain is recognized as a complex condition involving neurobiological adaptations rather than as simply an ongoing symptom of structural tissue damage [1]. The International Association for the Study of Pain (IASP) and the World Health Organization International Classification of Diseases 11th Revision (ICD-11) define chronic pain as pain that persists or recurs for longer than three months [1]. Epidemiological studies estimate that chronic pain affects 20–30% of adults globally, serving as a primary cause of disability, lost productivity and healthcare utilization [2,3]. Importantly, ICD-11 distinguishes between chronic primary pain (chronic pain is considered a disease in its own right, characterized by significant emotional distress or functional disability not better explained by another chronic condition) and chronic secondary pain (pain that originates as a symptom of an underlying disease process, such as chronic cancer pain, post-surgical pain or chronic inflammatory joint disease) [1].

Traditional primary care approaches to chronic pain often overemphasized the search for structural peripheral abnormalities or relied on escalating pharmacological analgesia. However, contemporary pain science demonstrates substantial clinical heterogeneity among patients with identical diagnostic labels [1]. Biological alterations—such as peripheral sensitization, central nervous-system hyperexcitability and impaired descending pain inhibition—interact dynamically with psychological characteristics (depression, anxiety, catastrophizing and fear-avoidance beliefs) and social determinants of health (socioeconomic status, occupational demands, health literacy, and systemic access constraints) [4].

To reflect these complexities, clinical terminology must be precise. While the term “precision medicine” strictly implies stratification based on validated, objective biological, molecular or genomic biomarkers predicting treatment response, current pain management relies primarily on individualized or personalized clinical care [1,5]. This involves tailoring care to an individual’s clinical phenotype, physical examination findings, psychosocial context, comorbidities and preferences.

Furthermore, while primary care family physicians are theoretically well positioned to manage chronic pain due to longitudinal doctor–patient relationships and holistic patient knowledge [6], substantial system-level barriers exist. Primary care providers routinely face tight 15-minute consultation windows, administrative demands, diagnostic uncertainty, limited dedicated pain training and fragmented access to allied health services like physiotherapy and psychology [6,7].

This narrative review presents a practical, balanced framework for individualized chronic pain management tailored to the constraints of primary care. It outlines efficient clinical assessment, mechanistic phenotyping, contemporary non-pharmacological and pharmacological evidence, a dynamic decision-making workflow and pragmatic implementation strategies.

### Methods

A structured literature search was conducted across MEDLINE/PubMed, the Cochrane Database of Systematic Reviews and major international health guideline repositories (including IASP, NICE, and CDC) for literature published from 2015 to 2026. Search strategies utilized combinations of Medical Subject Headings (MeSH) and free-text terms, including: chronic pain, primary care, pain phenotyping, nociplastic pain, neuropathic pain, biopsychosocial model, opioid stewardship, pain neuroscience education and implementation science.

Articles were selected for inclusion based on clinical relevance to primary care practice and rigor of the methodology, with preference for high-quality systematic reviews, meta-analyses, consensus guidelines and major clinical trials. References were qualitatively analyzed and synthesized to build a practical, evidence-informed clinical framework.

## 2. Discussion

### 2.1. Personalized Assessment and Pain Phenotyping in Primary Care

Individualized pain management begins with a targeted clinical assessment that integrates history, physical examination and appropriate diagnostic workups to identify contributing pain mechanisms rather than searching endlessly for structural abnormalities [1,8]. A central implication of the contemporary classification of chronic pain is that the clinician should move beyond a disease-label approach and instead determine which mechanisms are most relevant to the individual patient. Chronic pain may persist because of ongoing tissue pathology, dysfunction or injury within the somatosensory system, altered nociceptive processing or a combination of these processes [1,8]. This distinction is particularly important in primary care, where patients frequently present with several overlapping conditions and where imaging findings may not correspond closely with symptom severity or functional impairment.

The biopsychosocial model provides a practical framework for integrating these domains. Biological contributors include tissue pathology, neurological injury, inflammation, sleep disturbance and physical deconditioning. Psychological and social factors may also influence symptom persistence, coping, treatment engagement and functional recovery [4]. The purpose of this approach is not to imply that pain is psychological or that structural pathology is unimportant. Rather, it recognizes that chronic pain is multidimensional and that the relative contributions of different mechanisms can change over time. A patient initially presenting with an acute nociceptive injury, for example, may subsequently develop movement avoidance, sleep disruption, anxiety about activity and persistent sensory amplification. Management therefore needs to evolve as the clinical phenotype evolves.

### 2.2. Mechanistic Phenotyping and Overlap

Mechanistically, chronic pain is categorized into three primary descriptors, recognizing that these represent mechanistic contributors rather than mutually exclusive diagnostic categories as explained in Table 1 [1,8]:

- The nociceptive pain mechanism: arises from actual or threatened damage to non-neural tissue and is due to the activation of nociceptive neurons [1]. It is typically localized, proportional to tissue mechanical stress or inflammation, and is common in osteoarthritis and under active mechanical strain [1,9]. The clinical relevance of identifying nociceptive features is that interventions directed at the underlying tissue or inflammatory process may be appropriate. Nevertheless, even when nociceptive pathology is present, the intensity and persistence of chronic pain may be influenced by sleep, mood, physical activity, expectations and nervous-system sensitivity. Thus, identification of a nociceptive contributor should not automatically lead to escalation of pharmacological or procedural treatment.
- The neuropathic Pain Mechanism is caused by a lesion or disease of the somatosensory nervous system [1,11]. Symptoms include burning, lancinating or electric-shock sensations, dermatomal/anatomic neuro-distribution, hypoesthesia and allodynia [1,11]. Neuropathic phenotyping is particularly useful because several pharmacological approaches have evidence in appropriately selected neuropathic pain conditions [11]. However, the presence of a description such as “burning” alone should not be considered sufficient evidence of neuropathic pain. Clinical history needs to be integrated with neurological examination and, where appropriate, objective evidence of a relevant lesion or disease.
- The nociplastic pain mechanism is defined by the IASP as pain arising from altered nociception despite no clear evidence of actual or threatened tissue damage causing the activation of peripheral nociceptors, as well as a lack of evidence of disease/lesion of the somatosensory system [1,8]. Nociplastic features may be especially relevant in patients with persistent widespread symptoms, disproportionate symptom burden, sensory hypersensitivity and functional limitations that are not adequately explained by tissue pathology. Recognition of this phenotype can help clinicians avoid repeated diagnostic procedures and unnecessary escalation of analgesic therapy.
- The conceptual distinction: nociplastic pain is a clinical mechanistic descriptor (seen in fibromyalgia, complex regional pain syndrome type 1 and primary chronic low back pain) [1,8]. It should not be confused with central sensitization, which is an operational neurophysiological definition (the amplification of neural signaling within the central nervous system that results in pain hypersensitivity) [8]. Central sensitization is a neurobiological mechanism that may contribute to nociplastic pain, but the two terms are not synonymous [8].

This distinction has practical implications for communication. Patients may interpret an explanation involving altered pain processing as meaning that their symptoms are imaginary or that clinicians have failed to identify a “real” disease. Effective communication should instead emphasize that altered pain processing represents a biological phenomenon and that pain can remain genuine and disabling even when tissue damage is no longer the dominant driver. Such explanations can support engagement with rehabilitation while avoiding the false dichotomy between “physical” and “psychological” pain.

In clinical practice, many patients present with mixed pain mechanisms [1]. For instance, a patient with chronic radicular back pain may demonstrate nociceptive discogenic pain, neuropathic radiculopathy and secondary nociplastic central amplification [1,8]. Mixed phenotypes explain why a single intervention frequently produces incomplete benefit. They also support the concept of multimodal care, in which treatment components are selected according to the different contributors identified during assessment. Importantly, phenotyping should remain dynamic rather than being treated as a permanent diagnostic label. Changes in neurological findings, activity, sleep, mood, disease activity or tissue pathology can alter the dominant clinical mechanism over time.

### 2.3. Integrating Physical Examination and Ancillary Investigations

Clinicians should avoid managing chronic pain solely through subjective interview; direct physical examination is essential [1,8]. Examination provides an opportunity to determine whether the patient’s reported symptoms correspond to identifiable sensory, motor, musculoskeletal or inflammatory findings. It also helps establish a baseline against which subsequent functional and neurological changes can be evaluated.

- Physical Examination: IASP criteria require demonstration of sensory abnormalities to support mechanistic phenotyping [1,8]. Clinicians should evaluate for hyperalgesia (exaggerated pain response to normally painful stimuli) and allodynia (pain due to a stimulus that does not normally provoke pain, such as light touch or clothing) [8]. If neurological deficits or allodynia follow a precise neuroanatomical distribution (e.g., single dermatome or peripheral nerve supply), a neuropathic mechanism is supported; widespread sensory changes outside anatomical boundaries suggest nociplastic features [1,8]. A structured examination can therefore help distinguish focal neurological disease from more diffuse sensory amplification. Examination should also assess gait, strength, range of motion, balance, functional movement and signs of inflammatory disease when clinically indicated.
- Ancillary Investigations: Diagnostic imaging, electrodiagnostics (EMG/NCS) and laboratory testing (e.g., ESR, CRP, and the rheumatoid factor) should be used judiciously to rule out secondary red flags, inflammatory arthropathies or structural compressive radiculopathies. If a patient with widespread pain displays significantly elevated CRP and RF, care must adapt away from primary nociplastic management toward rheumatologic co-management for inflammatory disease. Once serious secondary causes are ruled out, routine repeat imaging for non-specific chronic pain should be avoided to prevent over-medicalization. This approach is particularly important because incidental structural findings can reinforce a patient’s belief that ongoing tissue damage is responsible for every symptom and may promote repeated interventions without corresponding functional improvement.

The role of testing should therefore be problem-oriented rather than reassurance-oriented. Imaging is most useful when the result is expected to alter diagnosis, prognosis or management. Similarly, electrodiagnostic testing may be useful when the clinical examination raises uncertainty about the presence, distribution or severity of a peripheral neurological process. In contrast, indiscriminate testing can increase incidental findings and contribute to diagnostic cascades.

### 2.4. Primary Care Feasibility and Goal Setting

Primary care clinicians often express concern that a “comprehensive biopsychosocial assessment” is unfeasible in a standard 15-minute consultation [6,7]. Rather than conducting an exhaustive evaluation in a single visit, primary care assessment can be operationalized using a staged, longitudinal evaluation across multiple brief encounters [6]. Focus should be placed on identifying specific red flags (malignancy, infection, and progressive neurological deficit) and establishing patient-defined functional goals (e.g., walking 20 minutes daily, improving sleep continuity, or returning to part-time work) rather than solely scoring pain intensity on a 0–10 scale [5,16].

A longitudinal model also allows clinicians to observe how symptoms respond to changes in activity, sleep, medication exposure and rehabilitation. This may provide more clinically meaningful information than a single cross-sectional assessment. Functional goals should be specific, realistic and measurable. For example, “reduce pain” can be reframed as “walk for 15 minutes without stopping”, “resume household activities” or “improve sleep continuity”. Such goals allow both the clinician and patient to determine whether a treatment is producing meaningful benefit, even when pain intensity remains present.

The staged approach also makes implementation more realistic. One visit can focus on diagnostic clarification and red-flag screening, another on rehabilitation and education, and subsequent encounters on medication review and functional progress. This distributes the biopsychosocial assessment over time, without requiring primary care clinicians to reproduce a specialist pain consultation during every encounter [6,7].

#### 2.4.1. Personalized Non-Pharmacological Management

Non-pharmacological strategies form the foundation of chronic pain care and should be introduced prior to or alongside selective pharmacotherapy as described in Table 2 [5,16]. The emphasis on active rather than passive care is particularly relevant because chronic pain frequently becomes associated with reduced activity, fear of movement and progressive physical deconditioning. These processes can reinforce disability even when the original tissue pathology has stabilized.

#### 2.4.2. Exercise and Physical Rehabilitation

Exercise therapy is supported by high-quality evidence across chronic low back pain, osteoarthritis and fibromyalgia [10,17]. To optimize adherence, primary care clinicians should collaborate with physiotherapists, occupational therapists or exercise physiologists [6]. Exercise prescriptions must be individualized according to physical capacity and fear-avoidance beliefs, prioritizing gradual aerobic activity, strength training or movement re-education [10]. Adherence can be monitored during follow-ups via simple self-report logs, step-trackers or wearable activity monitors [19].

Importantly, exercise should not be presented as a test that patients must pass before they deserve additional treatment. Instead, activity should be progressively titrated to individual capacity. Patients with substantial fear avoidance may initially require very small increases in movement. The objective is to build confidence and function while avoiding unnecessary symptom flares that can reinforce avoidance. Over time, improvements in strength, endurance and activity tolerance may become more clinically meaningful than a modest numerical change in pain intensity.

Exercise selection should also reflect comorbidities and patient preference. Aerobic activity may be appropriate for one patient, while strengthening, graded movement or occupational adaptation may be more relevant for another. This reinforces the broader principle that personalization concerns not only the choice of treatment but also its intensity, delivery method and progression.

#### 2.4.3. Pain Neuroscience Education (PNE)

PNE educates patients on the neurobiology of chronic pain, explaining that persistent pain reflects nervous-system hypersensitivity rather than ongoing structural damage [13,14]. However, PNE should not be delivered as a standalone intervention or assumed to be a universal cure [13,14]. Explaining central sensitization does not automatically eliminate pain; PNE is most effective when integrated into a broader active physical rehabilitation strategy to reduce catastrophizing and facilitate engagement in movement [13,14].

The clinical value of PNE therefore lies partly in changing the patient’s interpretation of symptoms. Patients who interpret every painful sensation as evidence of injury may avoid movement, progressively reduce activity and become increasingly focused on symptom monitoring. Education can provide an alternative framework in which symptoms are acknowledged while movement and functional restoration remain possible. This communication strategy should be individualized and should avoid overly simplistic explanations suggesting that all chronic pain is generated centrally.

The “Learn and Move” approach further illustrates the potential value of combining education with active movement rather than treating education as an isolated classroom intervention [14]. In primary care, brief educational messages can be reinforced repeatedly during follow-up encounters, allowing patients to connect the concepts with their own activity and recovery.

### 2.5. Psychological Interventions

Cognitive Behavioral Therapy (CBT), Acceptance and Commitment Therapy (ACT) and Pain Reprocessing Therapy (PRT) have established clinical trial evidence in chronic pain [15]. These modalities help patients modify maladaptive beliefs, increase psychological flexibility and unlearn threat associations [15]. In time-limited primary care encounters, clinicians can apply CBT principles—such as goal setting, activity pacing and the addressing of fear avoidance—rather than attempting formal psychotherapy [15].

The role of psychological interventions should be framed as part of comprehensive pain treatment rather than as evidence that symptoms are psychological. Psychological distress, catastrophizing and fear avoidance can influence disability and treatment engagement without being the original cause of pain. Consequently, identifying these factors provides additional treatment targets. When more intensive therapy is appropriate, referral to a clinician trained in behavioral pain management may be necessary.

ACT may be particularly relevant when complete symptom elimination is unrealistic. By emphasizing valued activity and psychological flexibility, the approach can help patients pursue meaningful goals while pain remains present. CBT-oriented strategies such as pacing can similarly help patients avoid cycles of overactivity followed by prolonged symptom exacerbation.

### 2.6. Sleep and Lifestyle Interventions

Sleep disturbance impairs pain modulation and exacerbates central sensitivity [18]. Structured sleep assessment and sleep hygiene interventions (Cognitive Behavioral Therapy for Insomnia, CBT-I) are integral [18]. Lifestyle measures—including weight management, dietary adjustment, smoking cessation and stress management—further contribute to systemic inflammation reduction and functional recovery.

Sleep should be assessed routinely because it is both a potential contributor to persistent pain and an outcome that patients can recognize quickly. Improving sleep may enhance coping, energy and capacity to participate in rehabilitation, even when pain, itself, changes only modestly. Likewise, weight, smoking, nutrition and stress should be addressed and integrated into broader primary care management rather than presented as requirements that patients must satisfy before pain treatment can proceed.

### 2.7. Personalized Pharmacological Management

Pharmacotherapy should serve as an adjunct to active rehabilitation strategies rather than a standalone cure, as described in Table 3 [5,16]. Clinical selection must differentiate between chronic primary pain (where guidelines such as NICE NG193 explicitly discourage routine analgesic prescribing due to lack of efficacy and risk of harm) and chronic secondary pain (such as neuropathic pain or osteoarthritis, where targeted medications offer modest benefit) [5,16]. Clinicians must also distinguish between mechanism-informed treatment selection (choosing a class based on plausible pathways) and validated predictive matching (which remains an active area of ongoing clinical trial research).

This distinction is important because mechanistic phenotyping should not be interpreted as evidence that every patient with a particular phenotype will respond to a particular medication. The most appropriate approach is to identify a plausible indication, discuss the expected magnitude of benefit and adverse effects, establish a functional treatment target and reassess whether the medication is producing meaningful benefit.

### 2.8. Contemporary Pharmacological Evidence

- Acetaminophen (Paracetamol): Contemporary systematic reviews demonstrate limited to no efficacy for acetaminophen in chronic musculoskeletal conditions, including chronic low back pain and osteoarthritis [20]. It is not recommended for routine chronic primary pain [5]. Its use should be restricted to short-term, low-risk adjunct therapy in mild secondary musculoskeletal pain, taking into account liver function tests (LFTs) and a maximum daily dose of 3–4 g [20]. The limited efficacy of acetaminophen illustrates why medication continuation should be tied to demonstrated benefit rather than habit. If a patient cannot identify a meaningful improvement in activity or symptoms, continued long-term exposure should be reconsidered.
- NSAIDs: Oral NSAIDs provide modest short-term relief in chronic nociceptive/inflammatory pain (e.g., osteoarthritis) [9]. They are not recommended for chronic primary pain or nociplastic pain [5]. Prescribing requires individualized risk assessment regarding cardiovascular, renal and gastrointestinal comorbidities, using the lowest effective dose for the briefest appropriate duration [9]. Topical approaches may offer another means of limiting systemic exposure in selected musculoskeletal conditions. The overall objective remains the balancing of symptomatic benefit against cumulative adverse-event risk.
- SNRIs and Tricyclic Anti-depressants (TCAs): Duloxetine (SNRI) and low-dose TCAs (amitriptyline and nortriptyline) demonstrate efficacy in neuropathic pain conditions and fibromyalgia/nociplastic pain by modulating descending inhibitory pathways [11,12]. They are particularly useful when depression, anxiety or sleep fragmentation coexists [12]. Medication selection should nevertheless account for age, comorbidities, drug interactions, anticholinergic burden and tolerability. A theoretically appropriate medication may not be clinically appropriate if adverse effects interfere with rehabilitation or daily function.
- Gabapentinoids (Gabapentin/Pregabalin): Gabapentinoids are indicated for proven focal or central neuropathic pain (e.g., diabetic neuropathy, postherpetic neuralgia, radiculopathy with sensory findings) [11,21]. High-quality evidence does not support their generalized use for nonspecific chronic musculoskeletal or primary pain [21]. Off-label overprescribing in primary care should be avoided due to risks of sedation, gait instability and potential misuse [21]. Their continuation should therefore be reassessed when the original indication is uncertain or when functional improvement is absent.
- Opioids: Contemporary guidelines (e.g., CDC 2022 and NICE NG193) strongly advise against routine opioid therapy for chronic primary pain or nociplastic conditions due to limited long-term functional efficacy, hyperalgesia risks and dependence [5,16]. Opioids should not be framed as a solution for “severe refractory pain” in general. They may be considered only in carefully selected chronic secondary pain (or cancer-related pain) after thorough risk assessment and failure of safer alternatives [16,22]. The clinical focus should remain on function, safety and sustained benefit rather than escalation based solely on numerical pain scores.
  ○ Tapering Caution: When initiating an opioid taper for a patient on long-term therapy, fixed percentage schedules (e.g., “5–10% every few weeks”) must be avoided as a rigid rule [22]. Tapering must be highly individualized, slow and collaborative, adjusting the rate based on duration of use, dose, cardiac/psychiatric comorbidity, dependence and withdrawal symptoms [22]. The purpose of individualized tapering is not simply dose reduction but reduction in medication-related harm while maintaining patient engagement and access to appropriate non-opioid pain care. Abrupt or poorly supported reductions may undermine trust and worsen withdrawal-related symptoms.
- Interventional Non-Opioid Procedures: In specific secondary structural conditions, non-opioid procedures (e.g., joint injections, nerve blocks, radiofrequency ablation, or TENS) may be integrated as temporary adjuncts to facilitate physical therapy engagement. These interventions should be viewed within a broader treatment pathway rather than as substitutes for rehabilitation. Their value may be greatest when a temporary reduction in symptoms allows a patient to participate more effectively in movement-based care. Patient selection, expected duration of benefit and procedural risks should be explicitly discussed.

### 2.9. Shared Decision-Making, Dynamic Personalization, and Reassessment

**Figure 1:**
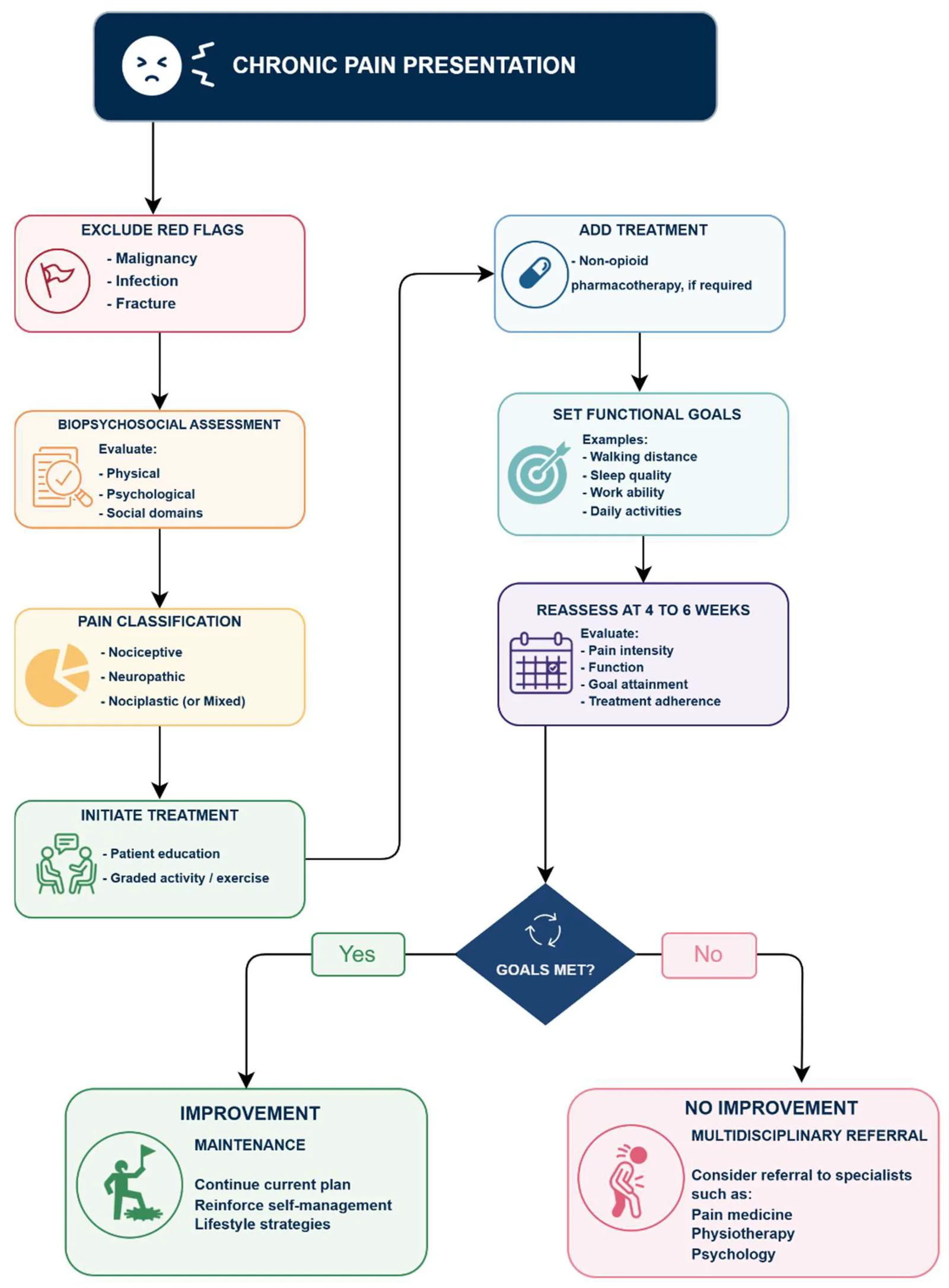
Clinical Pathway for the Evaluation and Management of Chronic Pain in Primary Care.

Personalized management requires a longitudinal, iterative framework centered on shared decision-making with the framework explained in Figure 1 [5,6]. The process should begin with agreement on the principal goals of care, followed by selection of interventions that are acceptable to the patient and clinically appropriate for the identified mechanisms. Treatment should then be evaluated according to predefined outcomes rather than continued indefinitely because it was initially prescribed.

Shared decision-making is particularly important when expected treatment effects are modest. Patients should understand that chronic pain interventions frequently produce partial improvement rather than complete elimination of symptoms. This allows treatment decisions to be based on realistic expectations and helps avoid repeated switching between therapies in search of a single definitive cure.

#### Response and Reassessment Framework

Reassessment should occur at defined intervals (typically every 4–8 weeks after initiating or modifying a strategy) [5]. Clinically meaningful response must be evaluated using broad multidimensional criteria:

- Pain and Function: The Brief Pain Inventory (BPI), Oswestry Disability Index (ODI), or Roland-Morris Disability Questionnaire.
- Goal Attainment: Progress toward specific, patient-defined functional targets.
- Quality of Life: Improvements in sleep continuity, mood and daily participation.
- Treatment Burden: Medication adverse effects, polypharmacy burden and financial constraints.

A multidimensional outcome strategy is particularly important because pain intensity may fluctuate independently of functional improvement. A patient who reports similar pain scores but is walking farther, sleeping better and returning to work may be experiencing clinically meaningful benefit. Conversely, a small reduction in pain accompanied by substantial sedation or loss of function may not represent treatment success.

Reassessment should therefore ask four practical questions: Is the patient functioning better? Is the intervention producing meaningful symptom improvement? Is treatment causing harm or excessive burden? Does the current plan remain consistent with the patient’s goals? These questions can be incorporated into routine primary care follow-up without requiring extensive specialized measurement at every visit.

If meaningful functional progress is achieved, the plan is maintained, transitioning emphasis toward long-term self-management [6]. If progress is lacking or adverse effects occur, ineffective therapies (especially analgesics) should be systematically de-prescribed, and secondary referral should be considered [5,6]. This “trial, measure and adjust” approach prevents passive continuation of ineffective treatments and creates a clear pathway for escalation when primary care management is insufficient.

### 2.10. Implementation Strategies in Primary Care

To successfully operationalize individualized pain care within primary care constraints, health-system factors must be addressed [6,7]. Implementation science frameworks such as the Consolidated Framework for Implementation Research (CFIR) and the Practical, Robust Implementation and Sustainability Model (PRISM) highlight the necessity of aligning clinical recommendations with local practice environments (Table 4) [23,24].

The importance of implementation is that evidence-based recommendations alone do not guarantee evidence-based care. A primary care clinician may recognize that exercise, behavioral interventions and medication optimization are appropriate but still face barriers, including limited appointment time, inadequate access to physical therapy, insurance restrictions, fragmented behavioral health services and competing clinical priorities. Implementation strategies must therefore be designed around the realities of the clinical environment.

CFIR provides a framework for considering characteristics of the intervention, inner and outer setting, individual clinicians and implementation processes [23]. PRISM similarly emphasizes the interaction between the intervention and the broader healthcare environment [24]. Applying these concepts to chronic pain means that interventions should be adapted without losing their essential therapeutic components. For example, a practice may use standardized EHR prompts, shared visits or remote monitoring while preserving individualized goal setting and reassessment.

#### Addressing System Constraints and Social Determinants

- Practice-Level Adaptations: Primary care practices can implement structured Electronic Health Record (EHR) templates to track functional goals, mechanistic phenotypes and monitoring parameters across brief visits [6]. Group medical visits (chronic pain shared appointments) offer an efficient platform for delivering pain neuroscience education and self-management support without overloading individual schedules [25]. Collaboration with clinical pharmacists can streamline medication reviews and tapering schedules [6]. Standardized templates may also reduce variation between clinicians and improve continuity when several members of the primary care team participate in management.
- Healthcare System Determinants: The feasibility of individualized pain care is heavily governed by health funding structures [6,7]. Fee-for-service models and privatized systems often under-reimburse time spent on complex biopsychosocial counseling and non-pharmacological care, whereas value-based or tax-funded healthcare systems (e.g., integrated NHS primary care networks) better facilitate multidisciplinary co-location [6]. Primary care providers must distinguish between individual patient-level factors and broader social determinants of health (e.g., housing instability, health literacy and regional access to physical therapy) when designing self-management plans [4,6].

Social determinants can directly influence whether a theoretically appropriate treatment is feasible. A home exercise program may be difficult for a patient without safe space for activity, while frequent physical therapy appointments may be unrealistic for someone with transportation or financial limitations. Similarly, medication choices can be influenced by cost, insurance coverage and ability to attend monitoring appointments. Personalized care therefore requires adaptation to the patient’s circumstances rather than simply providing a standardized treatment list.

The use of shared medical visits may be particularly useful because it allows education and self-management skills to be delivered efficiently while creating opportunities for patients to learn from others with similar functional challenges [25]. Such models can complement rather than replace individualized assessment. Patients with significant neurological deficits, inflammatory disease, complex medication issues or diagnostic uncertainty may still require individualized specialist assessment.

### 2.11. Future Directions in Primary Care Pain Management

While emerging technologies offer future promise, their applicability in routine primary care must be evaluated pragmatically:

- Digital Health and Remote Monitoring: Mobile applications, activity trackers and electronic patient-reported outcomes (ePROs) offer real-time tracking of movement and sleep, enhancing primary care follow-up between visits [19]. These technologies may allow clinicians to identify changes in function earlier and reduce reliance on retrospective recall during office visits. However, digital tools should simplify rather than increase the workload of clinicians and patients.
- Artificial Intelligence and Precision Rehabilitation: AI decision-support algorithms may eventually assist primary care clinicians in phenotyping and treatment selection; however, current systems require extensive validation to avoid bias and cannot replace therapeutic clinician–patient rapport. The greatest potential may lie in supporting rather than replacing clinical reasoning—for example, organizing longitudinal symptom data, identifying changes in functional trajectories or prompting reassessment of medication burden. Such systems will require careful validation because chronic pain is heterogeneous and algorithmic predictions may reproduce biases present in their training data.
- Biomarkers and Pharmacogenomics: Advanced neuroimaging, systemic inflammatory biomarkers and pharmacogenomic testing remain active areas of research. At present, evidence does not support their routine clinical use in primary care, where detailed clinical assessment remains the gold standard [1,8]. Future biomarkers may eventually improve identification of clinically meaningful subgroups, but their value will depend on demonstrating that biomarker-guided treatment actually improves patient outcomes compared with careful clinical phenotyping.

Overall, the future of primary care pain management is likely to involve increasing personalization without abandoning the fundamentals of clinical medicine. Digital monitoring, implementation science and emerging precision approaches may enhance decision-making, but the core process remains assessment of mechanisms, identification of functional goals, active rehabilitation, selective pharmacotherapy and repeated reassessment. The most practical model is therefore not a single intervention but a structured clinical pathway capable of adapting as the patient’s phenotype and goals change.

## 3. Conclusions

Chronic pain is a complex, heterogeneous condition requiring a transition from broad symptom suppression toward individualized, function-oriented care [1,5]. By incorporating clinical phenotyping, targeted physical examination, active non-pharmacological therapies, selective guideline-concordant pharmacotherapy and longitudinal reassessment, primary care clinicians can provide effective, sustainable chronic pain management within routine practice settings [5,6].

## Figures and Tables

**Table 1:** **Pain Phenotypes, Clinical Features,** **and Mechanism-Informed Approaches.**

Mechanism/Phenotype	Key Physical & Clinical Features	Common Clinical Examples	Mechanism-Informed Management Focus
**Nociceptive** [1]	Localized pain; proportional to movement/stress; no primary neurological deficit [1].	Osteoarthritis, mechanical joint pain, tendonopathy [1,9].	Active physical rehabilitation, exercise, biomechanical modifications, short-term NSAIDs if indicated [9,10].
**Neuropathic** [1,11]	Anatomical/dermatomal distribution; burning/electric shock quality; focal hyperalgesia, allodynia, or sensory loss on exam [1,11].	Diabetic neuropathy, postherpetic neuralgia, radiculopathy [11].	SNRIs, TCAs, gabapentinoids, specialized physical rehabilitation [11,12].
**Nociplastic** [1,8]	Widespread non-anatomical pain; altered sensory processing; associated with severe fatigue, sleep fragmentation, and cognitive symptoms [1,8].	Fibromyalgia, chronic primary low back pain [1,8].	Pain neuroscience education (integrated in rehabilitation), graded exercise, CBT/ACT/PRT, sleep optimization [12,13,14,15].
**Mixed** [1]	Coexistence of nociceptive, neuropathic, and/or nociplastic features [1,8].	Failed back surgery syndrome, complex radiculopathy with central sensitization [1,8].	Individualized multimodal rehabilitation combining targeted physical, psychological, and selective pharmacological therapies [5,6].

**Table 2:** **Multimodal Non-Pharmacological** **Interventions.**

Modality	Primary Target & Mechanism	Primary Care Clinical Delivery Strategy	Expected Outcome
**Exercise Therapy** [10,17]	Improves physical capacity, reduces fear-avoidance, enhances endogenous opioid/cannabinoid inhibition.	Collaborative prescription with physiotherapy; gradual aerobic, strength, or flex activities [6].	Improved mobility, reduced disability, enhanced function [12,16].
**Pain Neuroscience Education** [13,14]	Reconceptualizes pain threat value; addresses central hypersensitivity beliefs.	Brief, structured explanations delivered within an active physical rehabilitation plan [13,14].	Decreased catastrophizing, increased active participation [13].
**Psychological Strategies** [15]	Addresses maladaptive coping, threat appraisal, and emotional distress.	Formal CBT/ACT/PRT referral; brief primary care CBT principles (pacing, goal setting) [15].	Improved psychological flexibility and functional self-efficacy [15].
**Sleep Optimization** [18]	Restores sleep architecture and central descending inhibition.	Sleep hygiene evaluation, stimulus control, and referral for CBT-I [18].	Improved pain tolerance, reduced daytime fatigue [18].

**Table 3:** **Contemporary Pharmacological Evidence & Primary** **Care Recommendations.**

Drug Class	Pharmacological Examples	Dominant Indication/Mechanism	Primary Care Evidence & Guideline Recommendations	Major Contraindications & Safety Factors	Recommendation for Chronic Primary Pain?
**Acetaminophen**	Paracetamol	Minimal central/peripheral action.	Limited to no efficacy in chronic back pain or OA. Avoid routine reliance [20].	Hepatic impairment, severe alcoholism.	**No** [5]
**NSAIDs**	Ibuprofen, Naproxen, Celecoxib	Cyclooxygenase (COX) inhibition; anti-inflammatory.	Modest efficacy in secondary nociceptive/inflammatory pain. Lowest effective dose for shortest duration [9].	CKD, peptic ulcer disease, severe CAD/HF, advanced age.	**No** [5]
**SNRIs**	Duloxetine, Venlafaxine	Inhibits serotonin and norepinephrine reuptake.	Effective for neuropathic pain and nociplastic pain (fibromyalgia) [11,12].	Uncontrolled hypertension, severe hepatic impairment.	**Consider (Duloxetine)** [5]
**TCAs**	Amitriptyline, Nortriptyline	Inhibits monoamine reuptake; sodium channel blockade.	Effective for neuropathic pain and sleep disturbance in chronic pain [11]. Initiate at low bedtime dose.	Cardiac conduction defects, glaucoma, severe anticholinergic risk in elderly.	**Consider (Low dose)** [5]
**Gabapentinoids**	Gabapentin, Pregabalin	Subunit modulation of voltage-gated calcium channels.	Effective only for focal/central neuropathic pain [11]. Not effective for nonspecific back pain [21].	Renal clearance impairment (requires dose reduction), sedation/fall risk [21].	**No** [5,21]
**Opioids**	Morphine, Oxycodone, Buprenorphine	Mu-opioid receptor agonism.	Restricted to selected chronic secondary pain after rigorous risk stratification. Taper slowly and collaboratively [16,22].	Substance use disorder history, severe sleep apnea, concurrent benzodiazepine use [16].	**No (Strongly Avoid)** [5,16]

**Table 4:** **Practical Implementation Strategies for** **Primary Care Practices.**

Implementation Domain	Practical Strategy in Primary Care	Operational Impact
**Clinical Workflow** [6,23]	Utilize EHR templates for longitudinal goal tracking and mechanistic phenotyping [6,23].	Streamlining documentation in 15-minute consultations [6,7].
**Interprofessional Collaboration** [6]	Partner with clinical pharmacists for medication reviews and structured opioid tapering [6].	Enhances prescribing safety and reduces provider burden [6,22].
**Group Visits** [25]	Implement chronic pain shared appointments for PNE and lifestyle counseling [25].	Efficient delivery of education to multiple patients [25].
**Care Coordination** [6,24]	Establish standardized referral pathways to community physiotherapy and psychology services [6,24].	Minimizes care fragmentation and improves multidisciplinary care [6,24].

## Data Availability

The original contributions presented in this study are included in the article. Further inquiries can be directed to the corresponding author.
